# Caveolin-1 is a primary determinant of endothelial stiffening associated with dyslipidemia, disturbed flow, and ageing

**DOI:** 10.1038/s41598-022-20713-7

**Published:** 2022-10-24

**Authors:** Elizabeth Le Master, Amit Paul, Dana Lazarko, Victor Aguilar, Sang Joon Ahn, James C. Lee, Richard D. Minshall, Irena Levitan

**Affiliations:** 1 Division of Pulmonary and Critical Care, Department of Medicine, University of Illinois at Chicago, 909 South Wolcott Ave, Chicago, IL 60612-7323 USA; 2 Department of Biomedical Engineering, Department of Medicine, University of Illinois at Chicago, Chicago, IL USA; 3 Department of Pharmacology, Department of Medicine, University of Illinois at Chicago, Chicago, IL USA; 4 Department of Anesthesiology, Department of Medicine, University of Illinois at Chicago, Chicago, IL USA

**Keywords:** Endocytosis, Membrane biophysics, Cardiovascular biology, Ageing

## Abstract

Endothelial stiffness is emerging as a major determinant in endothelial function. Here, we analyzed the role of caveolin-1 (Cav-1) in determining the stiffness of endothelial cells (EC) exposed to oxidized low density lipoprotein (oxLDL) under static and hemodynamic conditions in vitro and of aortic endothelium in vivo in mouse models of dyslipidemia and ageing. Elastic moduli of cultured ECs and of the endothelial monolayer of freshly isolated mouse aortas were measured using atomic force microscopy (AFM). We found that a loss of Cav-1 abrogates the uptake of oxLDL and oxLDL-induced endothelial stiffening, as well as endothelial stiffening induced by disturbed flow (DF), which was also oxLDL dependent. Mechanistically, Cav-1 is required for the expression of CD36 (cluster of differentiation 36) scavenger receptor. Genetic deletion of Cav-1 abrogated endothelial stiffening observed in the DF region of the aortic arch, and induced by a high fat diet (4–6 weeks) and significantly blunted endothelial stiffening that develops with advanced age. This effect was independent of stiffening of the sub-endothelium layer. Additionally, Cav-1 expression significantly increased with age. No differences in elastic modulus were observed between the sexes in advanced aged wild type and Cav-1 knockout mice. Taken together, this study demonstrates that Cav-1 plays a critical role in endothelial stiffening induced by oxLDL in vitro and by dyslipidemia, disturbed flow and ageing in vivo.

## Introduction

Endothelial stiffness is a fundamental biomechanical property that reflects cellular elasticity and deformability, which is critical for basic cell functions such as the ability to respond to mechanical stimuli, cell–cell interactions and motility. Previous work from our lab has shown that exposing aortic endothelial cells to oxidized modifications of LDL (oxLDL) results in significant cell stiffening^1,2^. More recently, we demonstrated that uptake of oxLDL and oxLDL-induced endothelial stiffening depend on the hemodynamic environment and both are significantly enhanced in cells exposed to non-unidirectional (disturbed) flow (DF) as compared to unidirectional (laminar) flow (LF)^3^. Furthermore, we found that the elastic modulus (stiffness) of aortic endothelium in mice is significantly elevated in response to high fat diet-induced obesity^3^ and during ageing^4^. Here we test the role of caveolin-1 (Cav-1) in endothelial stiffening in vitro and in vivo.

Cav-1 is known to play a major role in endothelial mechanotransduction. Earlier studies established that a lack of Cav-1 impedes the ability of the endothelial cells to align in the direction of the flow^5^ and is associated with impaired flow-induced vasodilation and NO production^6^. Cav-1 was also shown to be necessary for flow-induced vascular remodeling^6^. More recently, it was recognized that caveolae serve as buffers against an increase in membrane tension and protect cells from mechanical injury^7–9^. Notably, genetic deletion of Cav-1 was shown to be athero-protective^10^, with the athero-protection conferred by multi-cellular mechanisms including those in endothelial cells^11^ and monocytes^12^. A lack of Cav-1 in endothelial cells resulted in reduced uptake of lipoproteins into the arterial wall^11^. Conversely, endothelial-specific over-expression of Cav-1 accelerated atherosclerosis^11^. Studies by Ramirez^13^, however, found that the protective effects of Cav-1 depletion are independent of eNOS activation and suggested that it could be attributed to the inhibition of trans-endothelial lipoprotein transport^13^. However, the impact of Cav-1-dependent lipoprotein uptake on endothelial biomechanics remained poorly understood.

In this study, we addressed a hypothesis that genetic deletion of Cav-1 might be protective against endothelial stiffening under pro-atherogenic conditions. This hypothesis is based on our previous findings that endothelial stiffness is significantly increased in cells exposed to oxLDL^1,2^, which were shown to be internalized upon receptor binding via caveolae-mediated endocytosis^14^. We also showed that oxLDL uptake in aortic endothelium and oxLDL-induced endothelial stiffening requires scavenger receptor (cluster of differentiation 36) CD36^15^, a receptor that is known to bind and internalize oxLDL^16^ and to reside in caveolae^17^. Furthermore, we found that aortic endothelium in vivo undergoes significant stiffening in the pro-atherogenic region of the aortic arch, which is exacerbated by high fat diet and abrogated in mice lacking CD36 suggesting that endothelial stiffening in vivo under dyslipidemic conditions is also mediated by the uptake of oxLDL^3^. Surprisingly, progressive stiffening of aortic endothelium with age is also dependent in large part on CD36, suggesting that it may also be attributed to oxLDL-induced endothelial injury^4^. Our current study demonstrates that Cav-1 plays a critical role in oxLDL-induced endothelial stiffening in vitro and in dyslipidemia and age-related endothelial stiffening in vivo indicating that this process is mediated by the CD36/Cav-1 pathway and suggesting a new mechanism for the athero-protective and possibly anti-ageing effects of Cav-1 depletion.

## Materials and methods

### Cellular models

WT, Cav-1 KO and CD36 KO MAECs were isolated in-house using five male mice (10 weeks old) from each strain of WT (C57BL6/J), Cav-1 KO (B6.Cg-Cav1^tm1/nls^) and CD36 KO (B6.129S1-Cd36^*tm1Mfe*^/J), all purchased from Jackson Lab. Aortas were extracted from each group and were digested in neutral protease/elastase (1 mg/ml each, Worthington) in HEPES buffer at 37 °C for 45 min. Collagenase Type IV (final concentration 6 mg/ml, Worthington) in HEPES buffer was added to the tissue with the digestion continuing for another 15 min. Purification of endothelial cells was performed with the MACS (magnetic-beads associated cell sorting) system from Miltenyi Biotech and followed the company’s recommended procedure. Briefly, digested cells were incubated with anti-mouse CD31 antibody conjugated to magnetic beads and sorted using a magnetic column. Antibody captured endothelial cells were plated on a gelatin coated dishes with confirmation by immunostaining for the endothelial markers PECAM-1 and vWF.

All MAECs (mouse aortic ECs) and MMVECs (mouse microvascular ECs) were cultured using Endothelial Cell Growth Medium (EGM-2, Lonza, Roche) supplemented with the EGM_TM-2_ BulletKitTM (Lonza) plus 10 μg/ml penicillin/streptomycin (Gibco; Grand Island, NY) and 2% FBS (fetal bovine serum, Invitrogen). MAECs were used at passages 4–10, and WT and Cav-1 KO MMVECs were used in experiments between passages 10–15. All cells were fed every 2–3 days, split every 3–7 days and maintained in a humidified incubator at 37 °C with 5% CO_2_.

#### Adenoviral overexpression of Cav-1

To overexpress Cav-1, we transfected an adenovirus, Cav-1-AD into endothelial cells, as previously described^18^. In brief, plated ECs at 70% confluence were transfected with Cav-1-AD and the media was changed the next day. Experiments were performed 48 h later.

### Mouse models

Wild Type (WT, C57BL/6) and Cav-1 KO (B6.Cg-Cav1^tm1/nls^) male mice were purchased from Jackson laboratories and bred in-house. Some cohorts of mice included both males and females. Euthanasia was performed with CO_2_ or isoflurane followed by cervical dislocation, except for the experiments involving blood collection. For these experiments, the mice were sedated using isoflurane, decapitated and exsanguinated. The blood was chilled on ice for approximately 30 min followed by centrifugation at 4 °C for 10 min at 1500 rpm. The top layer was collected for further analysis. *OxLDL quantification* was performed in-house using a sandwich ELISA (Elabscience Biotech) following the manufacturer’s protocol. All animal protocols were approved by and followed the guidelines established by University of Illinois of Chicago’s Institutional Animal Care and Use Committee (Protocol 19-163) and reported according to ARRIVE (Animal Research: Reporting of in vivo Experiments) guidelines.

#### Diet-induced hyperlipidemia

We used a diet-induced hyperlipidemic model, in which 8–10 week old WT and Cav-1 KO male mice were fed a high fat, high cholesterol diet (Harlan, TD.88137) for 1 month.

This diet consists of 0.2% total cholesterol and 21% total fat of which more than 60% is saturated (425 kcal from fat). Regular chow (RC) and HFD fed mice were weighed at the start and end of the diet period.

#### Mouse vessel extraction

Following euthanasia, the mouse aorta was extracted and cut longitudinally to reveal the endothelial monolayer of the descending aorta and the inner curvature of the aortic arch. These intact aortas were gently washed to remove excess blood. The live tissue samples were attached to glass coverslips, endothelial side up, using double sided tape (Dritz) and maintained in Hank’s buffering solution (ThermoFisher).

#### Denuding

The sub-endothelial layer was obtained as previously described^19^ and visualized using 3D rendered confocal imaging^4^. In brief, a cotton-tipped applicator was wetted with Hank’s buffered salt solution and gently rubbed on the endothelial layer ~ ten times and then washed to remove debris.

### Parallel plate flow apparatus

The parallel plate flow apparatus (Ibidi, Integrated Biodiagnostic, Munchen, Germany) was used to create physiologically relevant shear stress, as described previously^3,20^. Laminar and disturbed flow patterns are created in the same flow chamber by a step barrier midway through flow channel. The step barrier chamber is modeled after the device described in DePaola et al. in which a region of recirculating disturbed flow (DF) is generated by the step barrier across the flow channel but the pressure at the inlet of the chamber is the same as in the outflow^21,22^. The shear stress values in the DF recirculating regions of the flow chamber are lower but also characterized by high shear stress gradients, as compared to the laminar regions^22^ and is also reported for the atheroprone regions of the aorta (as reviewed by Ref.^20^). Using computational fluid dynamic simulations (Comsol Multiphysics 5.1, Comsol Inc., Burlington, MA) the height of the step barrier in the flow chamber was optimized to maximize the area of disturbed flow following the step, as previously described^3,22^. The step barrier chamber was fabricated at UIC’s Microfabrication Foundry (PI: David Eddington, PhD) with polydimethysiloxane (PDMS, Ellsworth Adhesives, Germantown, WI) using photolithography, a well-documented material and method for engineering microfluidic devices to use in biological applications. For each flow experiment, endothelial cells were exposed to physiological flow patterns for 48 h: 10–20 dynes/cm^2^ in the laminar regions and low (0–5 dynes/cm^2^) recirculating flow in the disturbed flow regions immediately following the step barrier.

### Atomic force microscopy microindentation

Cell stiffness was assessed by measuring the elastic modulus using an Asylum MFP-3D-Bio atomic force microscope (Santa Barbara, CA), as previously described^3^. Briefly, the elastic modulus (stiffness) is quantified by the indentation of the cell’s surface by a cantilever tip to produce force-distance curves. The cantilever descends towards the cell at 2 μm/s velocity until a 3 nN trigger force, corresponding to 0.5–1 μm indentation depth (10–20% of total cell height) is reached. For cultured ECs, stiffness measurements were acquired in the peri-nuclear region in 15–25 cells per condition per experiment. With tissue samples, the endothelial (or sub-endothelial) layer is exposed to obtain force-distance curves at 10–20 distinct sites throughout the tissue sample, excluding the edges.

The Asylum brand of AFM uses a silicon nitride cantilever with a 35° cone tip with the spring constant determined in each experiment (range of 0.08–0.24 N/m). The bidomain polynomial model was fit to the experimental force curve using a standard least-squares minimization algorithm.

Native oxLDL (50 µg/ml of medium oxidized, Alfa Asear, 1 h) was used for AFM stiffness measurements of cultured (non-flow treated) endothelial cells.

### Microscopy and immunohistochemistry

Images were captured using a Ziess LSM 880 confocal, Ziess 710 confocal or a Ziess Axiovert Fluorescent Microscope with a long-distance 40× lens.

*OxLDL uptake* was quantified by adding 1 µg/ml of fluorescent rhodamine labeled oxLDL (medium oxidized DiI (1,1′-dioctadecyl-3,3,3′,3′-tetramethylindocarbocyanine perchlorate)-oxLDL, Alfa Aesar) into the flow medium for the duration of the experiment (48 h). In all experiments involving oxLDL, FBS is substituted with 2% lipoprotein deficient serum (Sigma-Aldrich). After the cessation of flow, the cells were fixed and washed within the flow chamber: washed twice with PBS (with Ca/Mg), once with a 0.5 M NaCl 0.2 M acid wash buffer and twice with PBS. The intermediate wash step with the acid wash buffer ensures removal of any remaining extracellular DiI-oxLDL particles bound to the EC surface. Average cellular fluorescence was assessed using ImageJ by outlining 4–7 cells per image and 3–5 images per condition for each experiment. For experiments not involving flow, ECs were treated with 50 µg/ml DiI-oxLDL for 1 h, a dosage and time previously determined to induce EC stiffness^15^.

#### Immunostaining cells

ECs were seeded on sterile plasma treated MatTek Coverslip Dishes (MatTek Corp., P35G-1.5-14-C) so that they would be 70–80% confluent in 24 h. For all immunocytochemistry studies, after 24 h, ECs were fixed with 4% PFA in PBS (Electron Microscopy Sciences, 15700) for 10 min, permeabilized with 0.2% Triton-X 100 (Fisher Scientific, BP151-100) in PBS for 15 min, wash 3× with PBS and blocked for 30 min with 5.5% Donkey Serum in PBS (Millipore Sigma, D9663). ECs were then labeled with the CD36 primary antibody (1:200, R&D Systems, AF2519) overnight at 4 °C, washed 3× with PBS and then labeled with Alexa Fluor 488 Secondary antibody (1:200, ThermoFisher Scientific, A-11055) mixed with Alexa Fluor 647 Phalloidin (1:200, ThermoFisher Scientific, A22287) for 45 min at room temperature. Finally, cells were labeled with DAPI (1:3000, ThermoFisher Scientific, D1306) for 5 min and mounted on Glycerol in PBS (9:1 ratio).

#### *En face* immunostaining

Whole-mouse cardiac perfusion/fixation was performed by perfusing a 15 ml Heparin Saline solution (0.9% Saline, 10 units Heparin Sodium Salt per ml) at a rate of 90 ml/h via a syringe pump, followed by 15 ml chilled 2% paraformaldehyde (PFA) perfusion at the same rate. Perfusions were performed through the left ventricle of the heart (after severing the right atrium). The thoracic aortas were excised/isolated and then further fixed in chilled 2% PFA for 1 h at 4 °C with gentle agitation. Fixed tissues were then permeabilized for an hour at room temperature with gentle agitation (0.2% Triton X-100 in PBS, permeabilization buffer), and then blocked for an hour at room temperature with gentle agitation (5.5% donkey serum in permeabilization buffer). Primary antibodies for rabbit anti-mouse Cav-1 (Abcam, ab2910) and rat anti-mouse VE-Cadherin (BD Biosciences, 555289) were added at a 1:100 dilution to a solution containing 2.75% donkey serum in permeabilization buffer. The primary antibody solution was added and incubated overnight at 4 °C with gentle agitation. Aortas were then washed 3× with permeabilization buffer for 30 min each at room temperature with gentle agitation. Secondary antibodies for donkey anti-rabbit Alexa Fluor 488 (Thermo-Fisher, A21206) and donkey anti-rat Alexa Fluor 568 (Thermo-Fisher, A78946) were added at 1:100 dilution along with DAPI (Thermo-Fisher, D1306) at a 1:3000 dilution to a solution containing 2.75% donkey serum in permeabilization buffer. The secondary antibody solution was added and incubated for 3 h at room temperature with gentle agitation. Aortas were washed again 3× in permeabilization buffer followed by a final PBS wash for 30 min each at room temperature with gentle agitation. Aortas were finally cut and bisected en face along the direction of flow and mounted on No. 1.5 coverslips.

#### 3D rendering and quantification

All MatTek coverslip dishes with ECs and mounted *en face* aortas were scanned via a Zeiss LSM 710 Laser Scanning Confocal System using a 63× 1.46NA oil immersion objective. 3D Z-stacks were obtained using optimized Nyquist criterion. After scanning, the Z-stacks were computationally analyzed through a custom 3D image analysis platform consisting of 3D reconstruction, 3D deconvolution, 3D surface creation of CD36 or Cav-1 positive areas and feature extraction (integrated 3D fluorescence intensity) of those areas.

#### Endothelial alignment

EC alignment was measured by analyzing the brightfield images acquired from the Ziess fluorescent microscope with fixed cells exposed to flow and oxLDL. Using ImageJ software, the angle of the long axis of the cell was measured (0 degrees being the direction of flow, 3–5 cells/image, 4–5 images/condition for each of the independent experiments).

### Real-time PCR

RNA was purified from cultured cells harvested from whole mouse aortas. Two strains were utilized: germline knockout of Cav-1 (#004585; Cav1^tm1MIs^) and corresponding background wild-type (#000664; C57BL/6J), acquired from Jackson Laboratories. To purify RNA, we utilized the Direct-zol RNA MicroPrep kit (Zymo Research). Briefly, confluent 6-well plate cell cultures were lysed with TRI reagent and mixed 1:1 with 190 proof ethanol. During the purification process, the additional step of DNAse treatment was incorporated. RNA samples were analyzed in a NanoDrop 2000 (Thermo Fisher) for purity assessment (≥ 1.9 A_260/280_) and quantification. After collection of at least 200 ng of pure RNA, samples were converted into cDNA using the High Capacity cDNA Reverse Transcription Kit (Applied Biosystems) and prepared for RT-PCR using POWER SYBR Green MasterMix (Applied Biosystems). PCR was performed in a Viia 7 Real-Time PCR System (Applied Biosystems) using a 40 cycle set-up on 20 μl samples. Gene expression was quantified using the 2^−ΔΔCT^ method. GAPDH and UBB were used as housekeeping genes. Primer sequences used for mouse Cav-1, CD36, GAPDH and UBB were purchased from IDT’s PrimeTime catalog.

Primers:

- GAPDH—Mm.PT.39a.1;
- UBB—Mm.PT.58.29038744.g;
- CD36—Mm.PT.58.32162630;
- CAV-1—Mm.PT.58.8.8184281.

### Statistical analysis

Appropriate sample sizes for these studies were calculated using a power analysis. Each data set was tested for normality and equal variance before deciding on the most appropriate parametric or non-parametric ANOVA. One-way ANOVA, analysis of variance (α = 0.05) using non-parametric analysis was performed to detect the differences in mouse weights and plasma oxLDL. The integrated 3D fluorescence intensity data for CD36 was plotted and analyzed via ordinary one-way ANOVA. The integrated 3D fluorescent intensity data for Cav-1 was analyzed using two-way ANOVA, with Wilcox sign post hoc test. All other statistical analysis was performed by two-way ANOVA with replication (α = 0.05) and non-parametric analysis using GraphPad to determine statistical significance. For parametric data sets, equal variance was not assumed and an unbalance ANOVA or a standard Student’s t-test was used. A P-value less than 0.05 was considered statistically significant.

## Results

### Caveolin-1 is essential for oxLDL-induced EC stiffening

**Figure 1:**
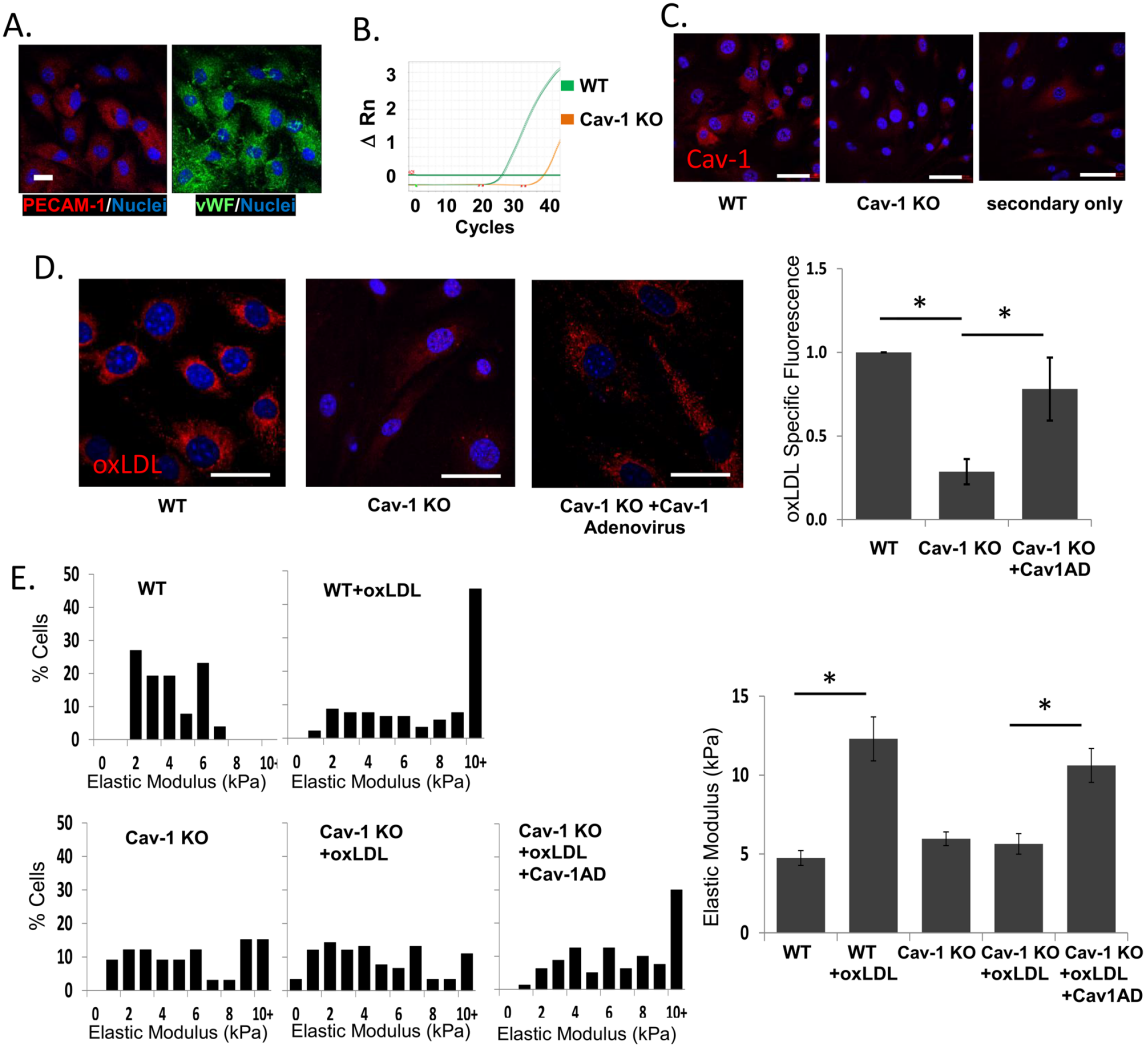
Caveolin-1 is required for oxLDL uptake and oxLDL-mediated stiffening in MAECs. (**A**) Representative confocal images showing the endothelial markers PECAM-1 and vWF in mouse aortic endothelial cells (MAECs). (**B**) Representative amplification profile of Cav-1 mRNA expression in mouse aortic endothelial cells (n = 4). (**C**) Representative confocal images of Cav-1 IHC staining in WT and Cav-1 KO ECs and Cav-1 rescue using Cav-1 adenovirus. (**D**) Representative confocal images of DiI-oxLDL uptake into WT, Cav-1 KO and Cav-1 adenovirus treated Cav-1 KO ECs (left) and summarized data of DiI-oxLDL uptake (right; mean ± SD; n = 5–8, 20–40 cells per experiment). (**E**) Histograms (left) and mean ± SD elastic modulus values (stiffness) of WT, Cav-1 KO and Cav-1 KO MAECs reconstituted with Cav-1 adenovirus after exposure to oxLDL or vehicle for 48 h (15–25 cells per condition/experiment, n = 4–10 independent experiments). Scale bars represent 20 µm. *P < 0.05 by ANOVA.

To test whether oxLDL-induced endothelial stiffening depends on Cav-1 expression in the aortic endothelium, primary mouse endothelial cells (MAECs) were isolated from aortas of WT and Cav-1 KO mice. Endothelial markers, PECAM-1 (platelet endothelial cell adhesion molecule) (Fig. 1A, left) and vWF (von Willebrand factor) (1A, right) confirm the purity of the MAECs isolation. Absence of Cav-1 expression was confirmed on the mRNA level using real time PCR (the ratio of Cav-1 mRNA in WT vs. Cav-1 KO cells being 1 vs. 0.001; representative real-time PCR profile is shown in Fig. 1B) and on the protein level by immunofluorescence (Fig. 1C). OxLDL uptake was assessed by exposing the cells to fluorescently labeled DiI-oxLDL following fixation and acid washed to remove any oxLDL particles adherent to the membrane surface. As expected, WT endothelial cells showed a clear punctate pattern of DiI-oxLDL, characteristic of a vesicular uptake mechanism, whereas deletion of Cav-1 resulted in a significant decrease in oxLDL uptake, which was fully rescued by infection of Cav-1 KO cells with an adenoviral vector expressing Cav-1 (Fig. 1D).

We also observed that genetic deletion of Cav-1 abrogated oxLDL-induced endothelial stiffening but had no effect on endothelial stiffness in the absence of oxLDL. A decrease in the elastic moduli in Cav-1 KO cells exposed to oxLDL is apparent from the leftward shift of the histogram of elastic moduli that shows the distribution of the moduli in individual Cav-1 KO cells as compared to WT endothelial cells. Similar to oxLDL uptake, the loss of oxLDL-induced stiffening in Cav-1 KO cells was fully reversible by Cav-1 reconstitution, which is apparent from the rightward shift of the histogram that matches the WT cells (Fig. 1E). These changes were further confirmed by comparing the average values for the elastic moduli for the five cell populations (Fig. 1E, right).

### Cav-1 regulates expression of CD36

**Figure 2:**
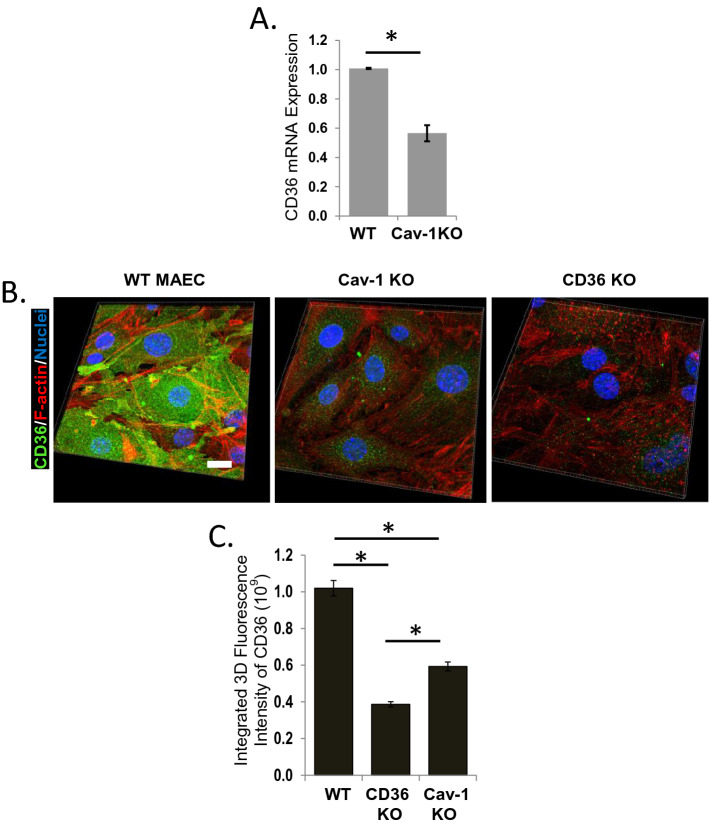
Cav1 regulates the expression of oxLDL scavenger receptor CD36. (**A**) Real-time PCR plot showing decreased CD36 mRNA expression in Cav-1 KO MAECs. (**B**) Representative 3D rendered confocal images showing CD36 expression in WT, CD36 KO and Cav-1 KO MAECs and (**C**) average integrated 3D fluorescence intensity of CD36 (n = 4, 5–8 cells per condition/experiment). Scale bars are 10 µm. *P < 0.05 by ANOVA.

We have shown earlier that scavenger receptor CD36 mediates endothelial uptake of oxLDL and oxLDL-induced endothelial stiffening^15^. CD36 is also known to reside in caveolae^17^ and has been shown to be significantly reduced in the aortas of caveolin-null mice, whereas increase in Cav-1 expression results in a concurrent increase in CD36 expression^10^. Consistent with the previous studies, we found that in Cav-1 KO MAECs, CD36 expression was significantly reduced at both the mRNA level, as assessed by real-time PCR (Fig. 2A) and on the protein level, as assessed by immunostaining and confocal imaging (Fig. 2B,C). Representative 3D rendered images of WT, Cav-1 KO and CD36 KO MAECs (Fig. 2B) and integrated fluorescence intensity of CD36-specific fluorescence (Fig. 2C) show that CD36 expression is significantly reduced in Cav-1 KO MAECs, as compared to WT cells, but not completely abolished, as evidenced by comparing CD36-specific fluorescence in Cav-1 KO and CD36 KO MAECs. These observations suggest that Cav-1 may regulate oxLDL uptake and oxLDL-induced endothelial stiffening, at least in part, via regulation of CD36 expression.

### Differential effects of laminar and disturbed flow on oxLDL uptake and endothelial stiffness are abrogated in the absence of Cav-1

**Figure 3:**
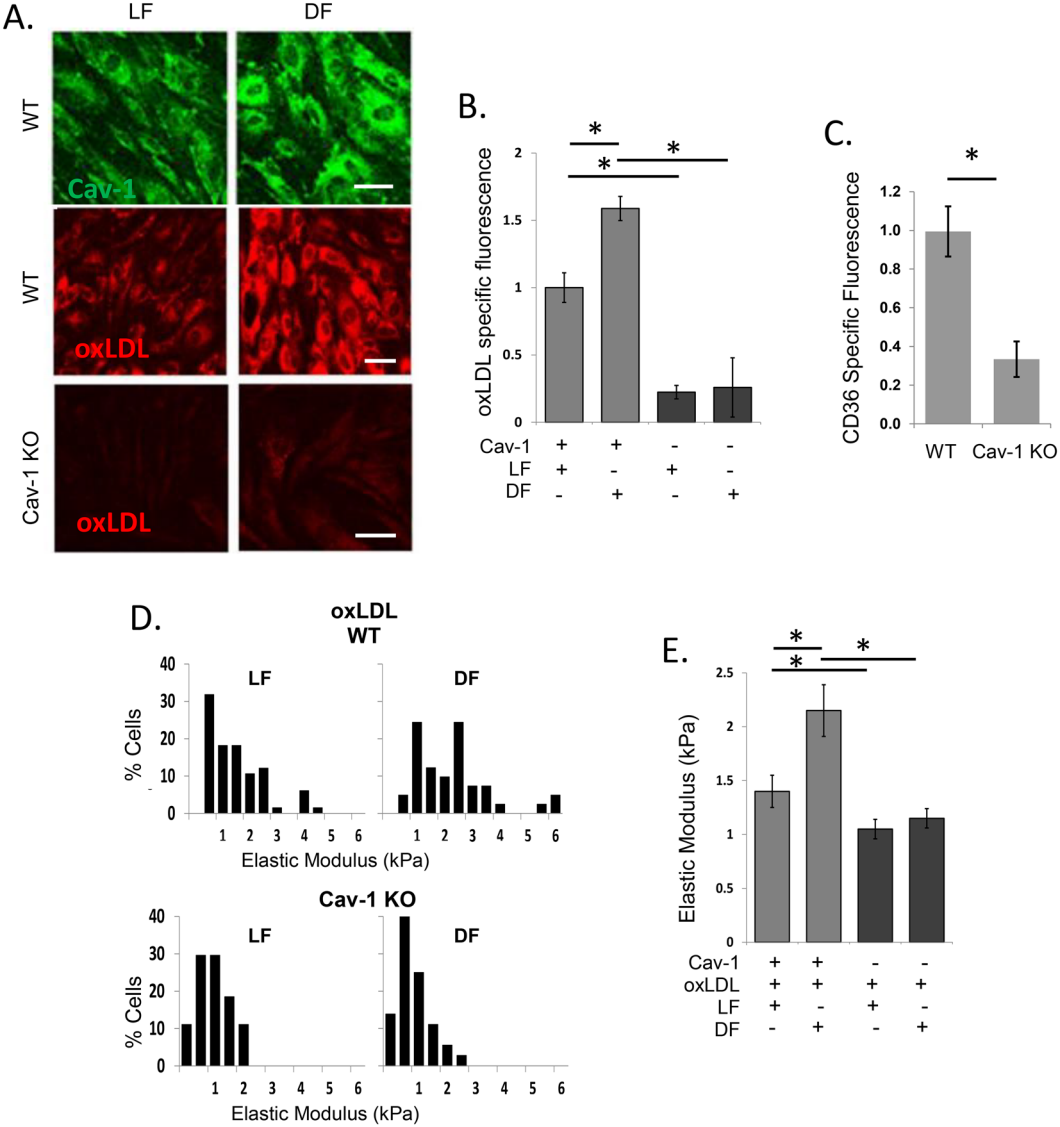
Disturbed flow-induced increase in oxLDL uptake and endothelial stiffening is mediated by Cav-1. (**A**) Representative confocal images of Cav-1 specific fluorescence (green, top) in WT ECs and DiI-oxLDL uptake (red) in WT (middle) and Cav-1 KO (bottom) in mouse microvascular ECs (MMVECs) exposed to laminar flow (LF) or disturbed flow (DF). Scale bars are 20 µm. (**B**) DiI-oxLDL uptake in Cav-1 KO and WT MMVECs (20–40 cells per condition/experiment; Mean ± SD, n = 6). (**C**) CD36 expression in WT versus Cav-1 KO MMVECs (n = 4). (**D**) Histograms of elastic modulus values from Cav-1 KO and WT ECs exposed to oxLDL (1 µg/ml, 48 h) during laminar and disturbed flow (15–25 cells per condition/experiment, n = 5) and (**E**) summarized (mean ± SD; n = 6) elastic modulus values for WT and Cav-1 KO MMVECs following 48 h exposure to laminar or disturbed flow in the presence of oxLDL. *P < 0.05 by ANOVA.

Next, we tested the effect of Cav-1 expression on oxLDL uptake and stiffening under different flow environments in pulmonary microvascular ECs that express high levels of Cav-1^17,23^. As described previously, cells were seeded into a microfluidic device with a step barrier that creates distinct regions of LF and DF environments of 10–12 dyne/cm^2^ or < 3 dyne/cm^2^, respectively, for 48 h in the presence or in the absence of 1 µg/ml DiI-oxLDL^3^. As expected, WT ECs aligned in the direction of flow under LF conditions, whereas Cav-1 KO ECs lose the ability to align to flow (Supplementary Fig. I). Our new data show that while the uptake of oxLDL in WT cells is enhanced under DF compared to LF conditions, which was accompanied by an increase in Cav-1 expression, in Cav-1 KO cells, the uptake was significantly reduced and no increase was observed under DF (Fig. 3A,B). These data indicate that Cav-1 plays a critical role in the regulation of oxLDL uptake in different flow environments. Additional studies investigating CD36 expression in microvascular ECs devoid of Cav-1 revealed that like large artery ECs, there is also a decrease in CD36 expression in Cav-1 KO ECs, as determined by immunohistochemical staining quantified by a plate reader (Fig. 3C).

The loss of Cav-1 expression also abrogated endothelial stiffening induced by DF in the presence of oxLDL (Fig. 3D,E). As described previously^3^, endothelial cells exposed to a local area of DF in presence of oxLDL are significantly stiffer than cells exposed to LF in the same chamber (Fig. 3D,E left). This effect was not observed in the absence of oxLDL (Supplementary Fig. II). We also observed that the stiffness of Cav-1 KO endothelial cells is lower than WT cells under both laminar and disturbed flow, an effect observed again only in the presence of oxLDL (Fig. 3D bottom, E right). Most importantly, endothelial stiffening induced by disturbed flow and oxLDL was abrogated in Cav-1 KO cells.

### Cav-1 deletion eliminates endothelial stiffening in intact aortic arches

**Figure 4:**
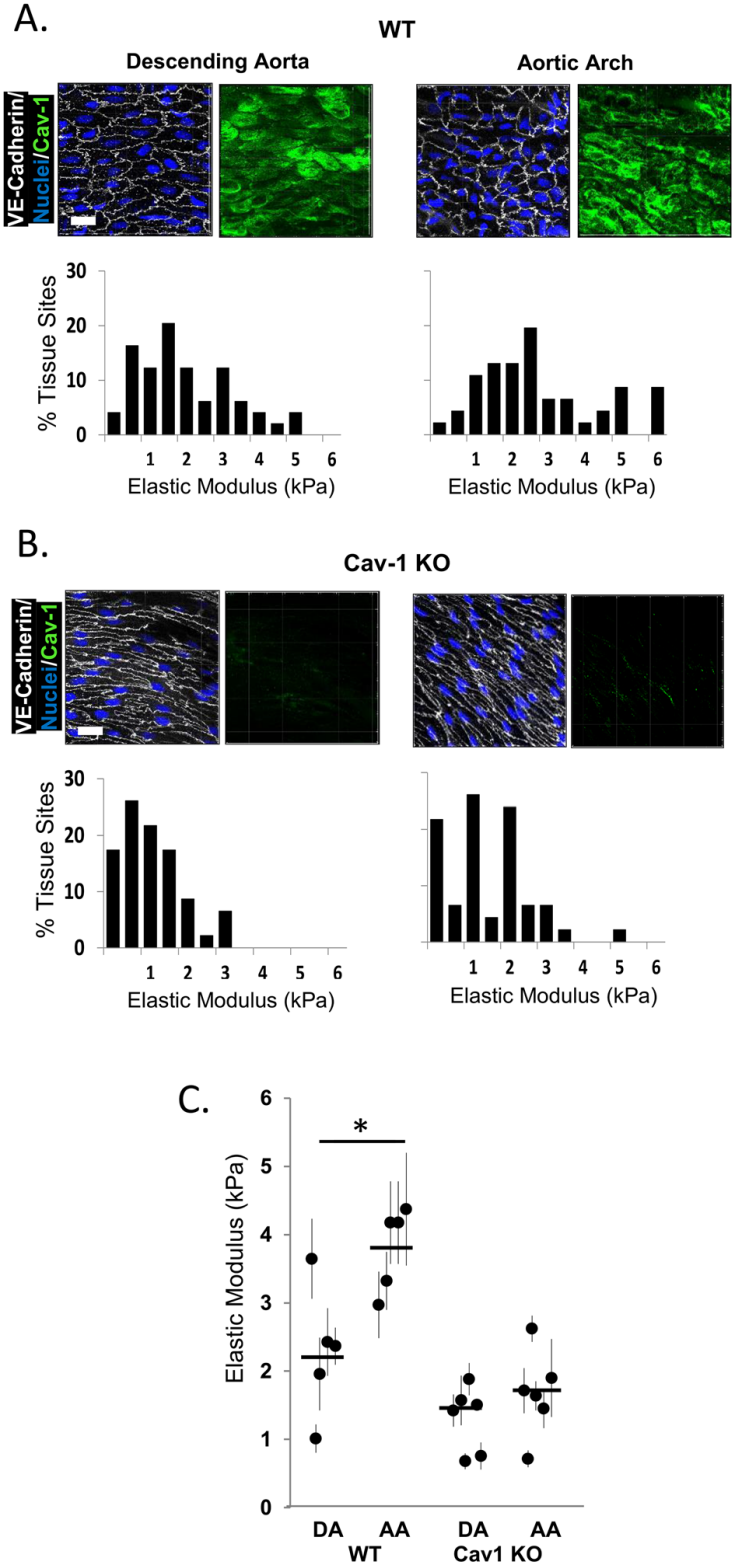
Absence of caveolin-1 abrogates endothelial stiffening in the pro-atherogenic aortic arch. (**A**) Histograms of elastic modulus values recorded from the descending aorta (left) and the aortic arch (right) of WT and (**B**) Cav-1 KO male 4 month old mice with representative confocal images to confirm lack of Cav-1 expression in Cav-1 KO mice (scale bars are 15 µm) and (**C**) data summary from AFM recording in (**A,B**) (Each circle is data from one mouse; error bars are SD from 10 to 15 measurements per aortic segment; horizontal line depicts mean of the total mice, n = 5–6 mice with 10–15 measurements per sample). *P < 0.05 by ANOVA.

The elastic modulus of the endothelial monolayer in vivo was assessed by AFM of freshly harvested intact mouse aortas, comparing the inner curvature of the aortic arch (AA) region, a segment known to be exposed to DF hemodynamic conditions, and descending aorta (DA) regions known to be exposed to laminar flow. Freshly-isolated vessels were opened longitudinally to expose the endothelial monolayer for the AFM probe, as described earlier^3^. The regions were identified by gross morphology and the presence of the intact endothelium was verified by *en face* staining of the aortas with endothelial marker VE-cadherin (see images in Fig. 4A,B, white). Cav-1-specific immunofluorescence is clearly visible in both AA and DA regions with no significant difference in Cav-1 expression between the two regions (see images in Fig. 4A, green). As expected, no Cav-1 staining was observed in aortas of Cav-1 KO mice (see images in Fig. 4B, green). As we described previously^3^, in WT mice the endothelial monolayer of the aortic arch along the inner curvature of the vessel was significantly stiffer than endothelium of the descending aorta from the same mouse, and we show here that this effect was completely abolished in Cav-1 KO mice (Fig. 4C). The histograms of the elastic moduli show aggregate AFM data from DA and AA regions for all WT mice and the difference in stiffness is again readily apparent from the rightward shift of the histogram (Fig. 4A, bottom right). Average elastic moduli values for individual mouse pairs also revealed a stiffer AA endothelium than the endothelium of the DA in four out of five pairs in the cohort (Fig. 4C). No difference in endothelial stiffness was observed between males and females, with both sexes having an increase in endothelial stiffness in the arch compared the DA (Supplementary Fig. IV). In contrast, the difference in endothelial stiffness between the DA vs AA was not observed in Cav-1 KO mice. Elastic moduli histograms showed no apparent shift and the averages for individual mice did not show changes between the two regions (bottom row of 4B, 4C). These experiments were performed with a cohort of 5–6 month old male mice and the AFM measurements of stiffness in WT and Cav-1 KO mice were done in parallel on the same day.

### Genetic deletion of Cav-1 protects against endothelial stiffening induced by high fat diet

**Figure 5:**
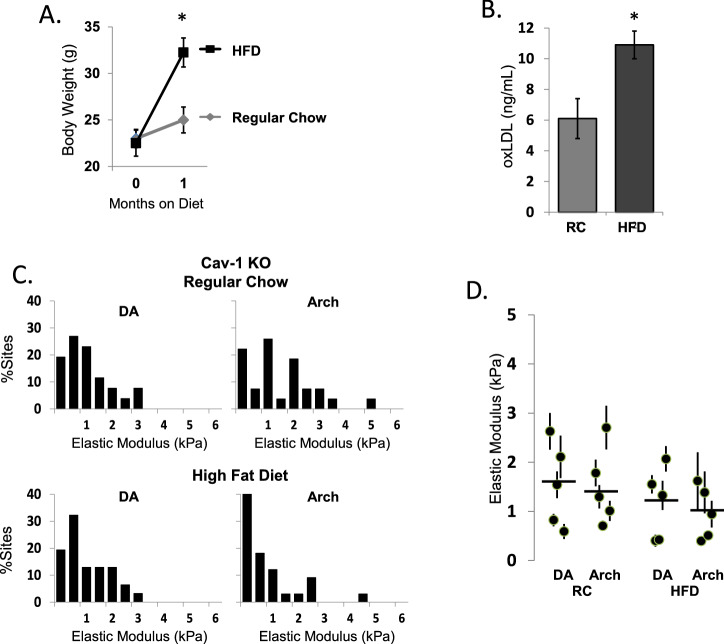
Absence of caveolin-1 abrogates endothelial stiffening induced by high fat diet. (**A,B**) Increase in body weight and plasma concentration of oxLDL in Cav-1 KO mice fed a HFD for 1 month. (**C**) Histograms of elastic modulus of DA and Arch in Cav-1 KO male mice fed regular chow (RC) or HFD for 1 month. (**D**) Summary of aortic elastic modulus recordings in Cav-1 KO mice. *P < 0.05 by ANOVA.

Our previous studies also showed that even a short treatment (4–6 week) with high fat diet (HFD) lead to significant stiffening of the aortic endothelium, as compared to regular chow (RC, low fat diet), most pronounced in the inner curvature of the arch (RC: DA 2.2 ± 0.2, AA 4.1 ± 1.4 kPa; vs. HFD: DA 4.9 ± 0.5, AA 8.9 ± 1.3 kPa)^3^. Here we show that this effect is fully abrogated in Cav-1 KO mice. In this experiment, mice also were maintained on high fat diet (42% of calories from fat) for 4–6 weeks, starting at the age of 8 weeks. Similar to the WT mice, Cav-1 KO mice gained more weight on HFD than on the regular chow diet (Fig. 5A) and had elevated levels of oxLDL in response to HFD (Fig. 5B). However, despite comparable increases in body weight and plasma levels of oxLDL, there was no increase in endothelial stiffness in Cav-1 KO mice exposed to HFD, neither in the DA, nor in the AA regions (Fig. 5C,D).

### Absence of Cav-1 mitigates endothelial stiffening with age

**Figure 6:**
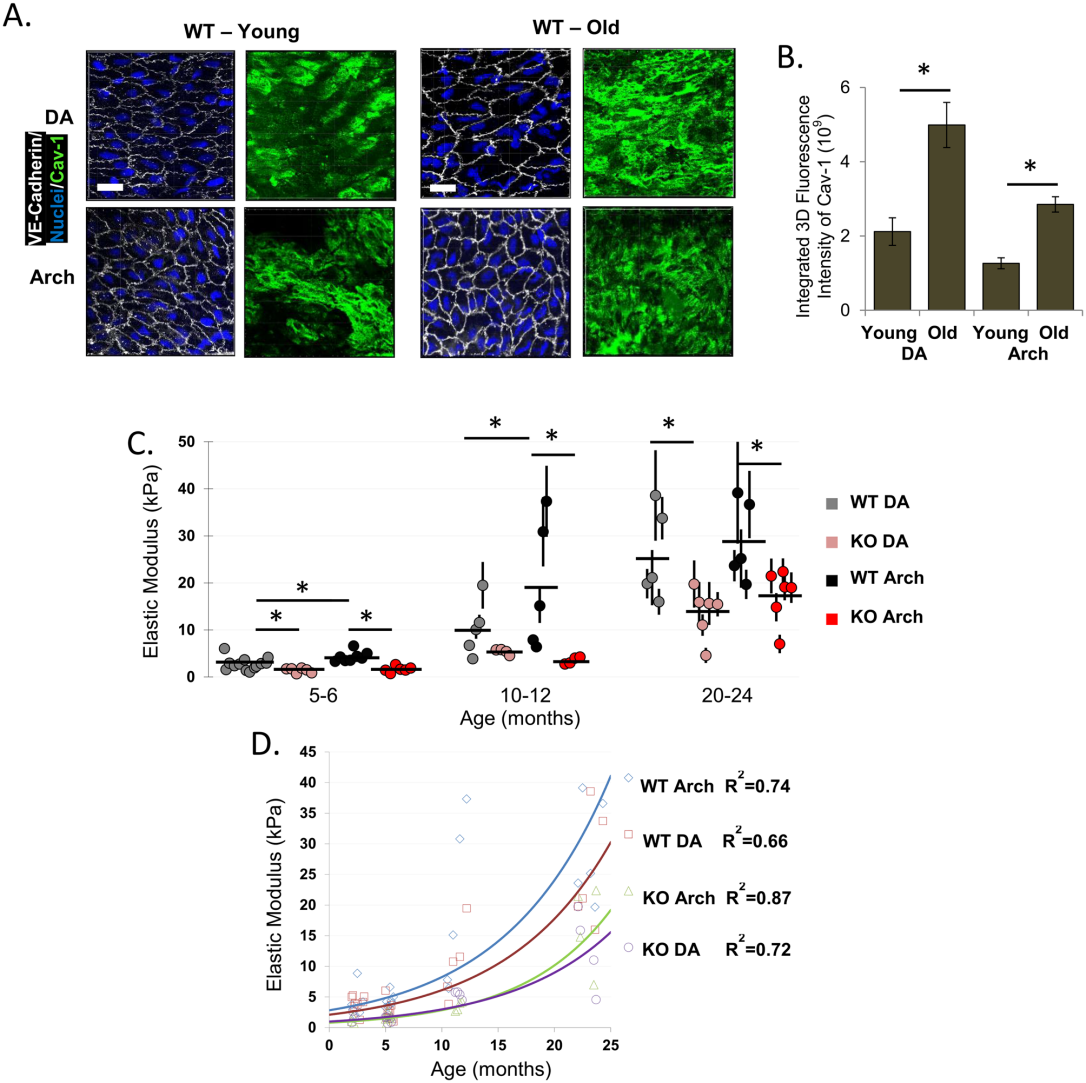
Age-associated increase in mouse aortic endothelial stiffening is mitigated by Cav-1 deletion. (**A**) Representative 3D confocal images (scale bars are 15 µm) of Cav-1 expression in the descending aorta and aortic arch in young (3–4 month old) and older (18–20 month old) WT mice (n = 3–4 mice per condition per age, 2–4 3D images per mouse) and (**B**) its quantification. (**C**) Summary of aortic elastic modulus values for WT and Cav-1 KO mice at each age. (**D**) Exponential curves representing mouse age versus elastic modulus. *P < 0.05 using ANOVA.

*En face* staining of Cav-1 of the endothelial layer of intact aortas isolated from young (3 month old) and aged (16–20 month old) WT mice revealed a significant increase in Cav-1 expression in the aortas of aged mice, as compared to aortas of the young animals (Fig. 6A,B). There is also increased heterogeneity and an apparent decrease in Cav-1 expression in the AA regions as compared to the DA regions, but no statistically significant difference between the two.

The elastic moduli of intact aortas in WT and Cav-1 KO mice was assessed in four age groups: juvenile (very young) mice 2–3 months old, young mice 5–6 months old, moderately aged mice 10–12 months old and advanced aged mice 20–24 months old. In all animals, the elastic modulus was assessed in the DA and in the AA regions. In WT mice, while there was no significant change in the elastic modulus of the aortic endothelium in mice between 3 and 6 months of age and then, there was a sharp increase (stiffening) in mice aged 10–12 months with further increase in 20–24 month old mice (Fig. 6C, the histograms are presented in Supplementary Fig. III). The increase in endothelial stiffness with age is observed in both DA and AA regions with the difference between the two regions maintained over the age range from young (2–3 months old) to moderately aged (10–12 months old), while in the most advanced age (20–24 months old) both regions become stiff with no difference between them.

In Cav-1 KO mice, ageing also resulted in an increase in endothelial stiffness of the aortas (Fig. 6C) but to a significantly lesser extent than in WT animals. In every age group and in both DA and AA regions, the stiffness of the aortic endothelium was significantly lower in Cav-1 KO mice, as compared in WT animals (Fig. 6C). Notably, the difference in endothelial stiffness between the DA and AA regions was abrogated in Cav-1 KO mice in all age groups.

To further quantify the impact of the loss of Cav-1 expression on age-induced increase in endothelial stiffness, the endothelial elastic modulus in DA and AA regions for each mouse at their exact age of use in all age groups (3, 6, 12 and 24 months old mice) were fitted with exponential curves that demonstrate the dramatic difference in stiffness between WT and Cav-1 KO mice over the life span (Fig. 6D). As expected, there was also a difference in the exponential coefficients between the DA and AA regions in WT mice, with the AA coefficient rising more steeply than that of the DA (2.8 vs. 2.1). In contrast, the stiffness of the aortic endothelium in Cav-1 KO mice was less steep and there was also less of a difference in the exponential coefficient between DA and AA regions (1.0 vs. 0.8).

**Figure 7:**
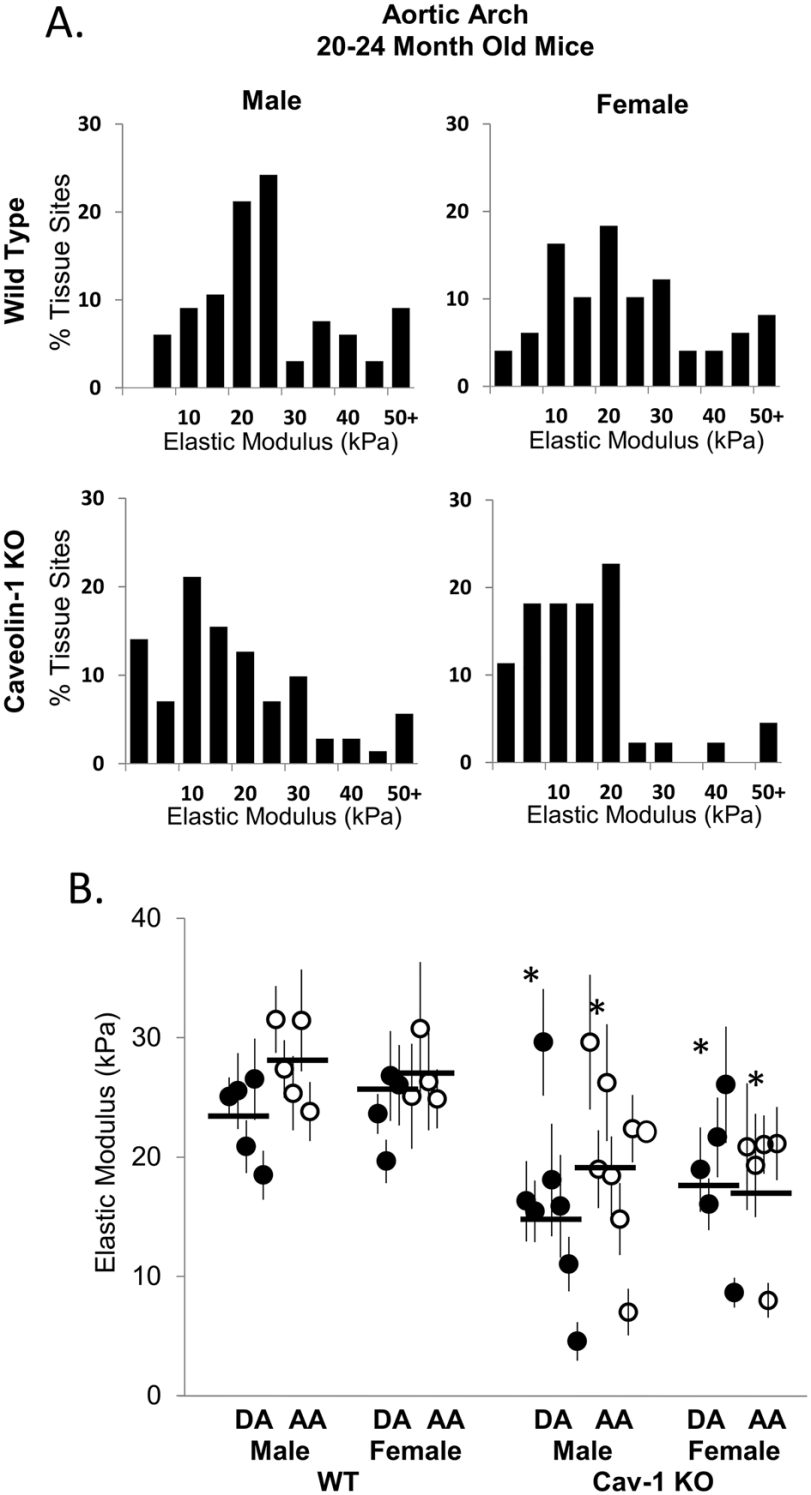
Cav-1 deletion in advanced aged mice reduces endothelial stiffening and no elastic modulus differences between the sexes. (**A**) Histograms of elastic modulus values from the aortic arch of advanced age (20–25 months) WT and Cav-1 KO males as compared to females with (**B**) Data summary of descending aorta and aortic arch from AFM recording in male and female WT and Cav-1 KO mice (n = 4–7 mice, 10–20 measurements per sample). *P < 0.05 compared to same vessel type and sex in WT mice as tested by ANOVA and a Student’s t-test.

Next, we determined if sex differences affected the stiffness of the endothelial monolayer in wild type and Cav-1 KO mice of advanced age (20–24 months old) (Fig. 7). These experiments were performed in separate cohort of aged mice with the elastic modulus measurements performed on a male/female pair in parallel, on the same day. Our data show that there is no difference in EC stiffness between males and females in either WT mice or in Cav-1 KO mice. Consistent with the previous cohort of male mice shown in Fig. 6, deletion of Cav-1 results in statistically significant lower elastic modulus of the endothelium in both male and female aged mice.

### Age-associated increase in aortic sub-endothelial layer stiffening is mitigated by Cav-1

**Figure 8:**
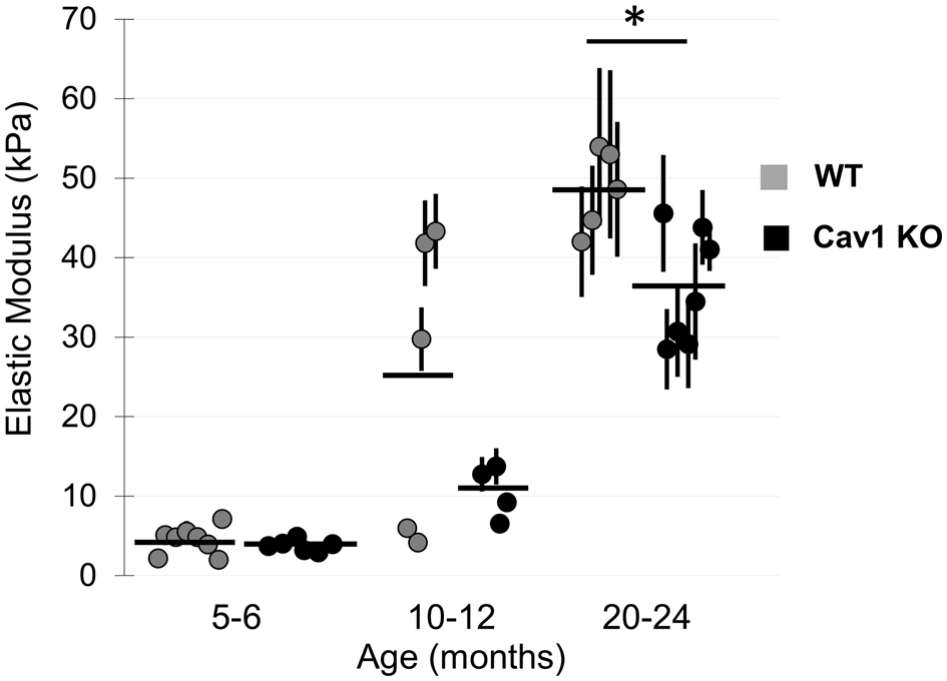
Age-associated increase in mouse aortic sub-endothelial layer stiffening is mitigated by Cav-1 deletion. Summary of sub-endothelial aortic elastic modulus recordings (average of DA and Arch) from WT and Cav-1 KO mice at three age groups. *P < 0.05 using ANOVA.

Finally, we also assessed the stiffness of the sub-endothelial layer of mouse aortas across age ranges between 5–6, 10–12 and 20–24 months (Fig. 8). As previously visualized and described^4^, obtaining the sub-EC layer was achieved by gently scraping off the endothelial layer and exposing the sub-endothelial layer to the AFM stylus. The sub-endothelial layer was significantly stiffer than the endothelial layer, particularly in 10–12 month old and 20–24 month old mice. Specifically, the average elastic modulus of the EC layer in the DA in advanced aged mice was 26 ± 9 kPa (Fig. 8B) vs. 55 ± 12 kPa in the sub-endothelial layer of the same aorta (Supplementary Fig. V). Notably, and in contrast to the endothelium, no significant difference was observed in the elastic moduli of DA and AA regions of the sub-endothelial layer (Supplementary Fig. V).

In terms of the effect of Cav-1 deletion on the sub-endothelial stiffness, it was age-dependent. In contrast to the endothelium, deletion of Cav-1 had no effect on the sub-endothelial stiffness in young and moderately aged mice (Fig. 8B), but resulted in mild sub-endothelium softening in the advanced aged mice. These observations suggest that while both endothelial and sub-endothelial age-related stiffening depends on Cav-1, the difference in stiffening between the DA and arch in the endothelium (Fig. 6B) is more sensitive to the loss of Cav-1 than it is in the sub-endothelial layer (Fig. 8B).

## Discussion

As increasing number of studies suggest that endothelial stiffening is a contributor to vascular dysfunction. Earlier studies showed that endothelial stiffness increases in the athero-prone region of the aortic arch as compared to the athero-resistant region of the descending aorta^3,24,25^. In addition, our studies showed that feeding mice high fat diet, even for a short period (4–6 weeks), significantly exacerbates endothelial stiffening, particularly in the arch^3^. Furthermore, deletion of the scavenger receptor CD36, which is known to be athero-protective^26^, abrogated an increase in endothelial stiffening in the aortic arch in mice fed regular chow and a high fat diet^3^. In this study, we addressed the role of Cav-1, a major transport protein regulating receptor-mediated endocytosis and transcytosis through the endothelium^27^, in regulating endothelial stiffness in cells exposed to oxLDL and different hemodynamic conditions in vitro and in athero-resistant and athero-prone regions of mouse aortas during the life span of the mouse. The main findings of this study are: (i) Cav-1 is essential for oxLDL-induced endothelial stiffening via regulation of oxLDL uptake in vitro and for high fat diet-induced hypercholesterolemia in vivo; (ii) Cav-1 plays a major role in endothelial stiffening observed in cells exposed to pro-atherogenic disturbed flow in the presence of oxLDL, which corresponds to endothelial stiffening in the atheroprone region of the aortic arch and finally (iii) Cav-1 plays a significant role in endothelial stiffening in ageing aortas. Taken together, our study demonstrates that Cav-1 is a master regulator of endothelial stiffness, particularly under pro-atherogenic conditions.

Moreover, this study provides new evidence that endothelial stiffening is pro-atherogenic by showing that genetic deletion of Cav-1, known to be athero-protective^10^, abrogates endothelial stiffening both in response to a pro-atherogenic hemodynamic environment and to a high fat diet. However, based on earlier studies that showed little or no difference in the blood pressure between WT and Cav-1 mice at the young age^28,29^, the difference in endothelial stiffness observed in our study does not translate into a measurable difference in the blood pressure. We propose that an athero-protective effect of Cav-1 deletion is its reduced endothelial stiffness and subsequent reduction in monocyte adhesion^30^, infiltration^31^ and differentiation into pro-inflammatory macrophages^12^. We also show here that the loss of Cav-1 is protective against endothelial stiffening during ageing, the latter well-known to cause dysfunction and exacerbate the development of cardiovascular disease.

This study also provides new evidence that endothelial and sub-endothelial stiffness are distinct, which is especially pronounced with advanced age. The endothelial layer is significantly softer than the sub-endothelium in 10–12 month old WT DA, for example 10.3 vs. 26.1 kPa, respectively, with the difference being statistically significant, which further increases in advanced age WT DA (25.8 vs. 53.0 kPa in EC and sub-ECs, respectively). The difference between aortic arch and descending aorta was also more pronounced in the endothelium, where it was observed in young and moderately aged mice, as compared to the sub-endothelium, where it was not observed. These observations suggest that endothelial stiffness is distinct and may be regulated independently from the stiffness of the underlying sub-endothelium layer.

Mechanistically, our observations suggest that the role of Cav-1 in determining endothelial stiffness is in regulating the uptake of oxidized lipids. Our key observation here is that the loss of Cav-1 leads to endothelial softening in the presence of oxLDL but has no effect in the absence of oxLDL. Similar to our earlier study, endothelial stiffening in response to disturbed flow in vitro was also is observed only in the presence of oxLDL and was abrogated by the deletion of Cav-1, whereas no effect of Cav-1 was observed on endothelial stiffness under flow in the absence of oxLDL. In both cases, a decrease in endothelial stiffness in Cav-1 KO cells was accompanied by a significant decrease in oxLDL uptake. These observations indicate that the mechanism by which Cav-1 regulates endothelial stiffness in vitro is by regulating the uptake of oxLDL. The connection between Cav-1, lipid uptake and endothelial stiffening in vivo is consistent with our findings that genetic deletion of Cav-1 abrogates endothelial stiffening induced by feeding mice a high fat diet, a model of mild hypercholesterolemia. The stiffening in the AA region of the aorta is also consistent with increased uptake of lipids in these regions^3^. It is also noteworthy that some naturally occurring lipoproteins, including oxidized LDL, are always present in the bloodstream and some accumulation of lipids in the AA occurs even in mice fed normal low fat diet^3^.

We found, however, no significant difference in Cav-1 immunostaining between the two regions indicating that the difference in endothelial stiffness between AA and DA cannot be attributed to an increase in Cav-1 expression but Cav-1 is generally considered to be abundant (not limiting) in endothelial cells. Taken together, our current data demonstrate that even though there is no increase in Cav-1 expression in the AA compared to the DA, its expression plays an essential role in the differential endothelial stiffness between the two regions because genetic deletion of Cav-1 results in both regions becoming softer and the difference between them disappears. In contrast, an increase in endothelial stiffness with age is accompanied with a strong increase in Cav-1 immunostaining, which is interpreted as an increase in Cav-1 expression. This is consistent with the Cav-1-dependent increase in endothelial stiffness in aged aortas but it does not prove that the stiffening is the result of increased Cav-1 expression, which still might not be a limiting factor. It is also possible that the stiffening is a result of a gradual process with a cumulative effect that develops over time. What our findings clearly show is that the absence of Cav-1 significantly attenuates age-related stiffening and that there are both a Cav-1-dependent and a Cav-1-independent age effects on EC stiffness.

In terms of the downstream signaling pathway, there is a general consensus that cellular stiffening in general, including endothelial stiffening, is a result of cytoskeletal remodeling^1^, primarily associated with activation of the RhoA (Ras homolog family member A) signaling pathway^15,25^ and the formation of stress fibers^32^. Our studies showed that endothelial stiffening is induced by oxidized lipids, specifically oxLDL, by activating the RhoA/MLCP (myosin light-chain phosphatase)/MLC (myosin light chain) signaling pathway and the uptake of oxLDL is mediated by CD36^15^. Here, we provide evidence that CD36/Cav-1-mediated endocytosis of oxLDL is responsible for the Cav-1-dependent stiffening effect. This notion is supported by a significant decrease in CD36 expression in Cav-1 KO aortas and by the similarities between the effects of Cav-1 and CD36 on endothelial stiffness across multiple conditions.

Finally, a critical question is what the role of endothelial stiffening is on vascular dysfunction. First, a contractile response that underlies endothelial stiffening is expected to contribute to the disruption of endothelial barrier integrity by applying force to the inter-cellular junctions and pulling them apart. Indeed, disruption of the endothelial barrier is a key early step in the development of atherosclerosis and also contributes to vascular dysfunction during ageing^33^. Disruption of the barrier is typically attributed to an inflammatory process initiated by the accumulation of the lipids in the vascular wall. Our findings provide evidence that disruption of the barrier by an endothelial contractile response/stiffening may be the result of a direct effect of oxidized lipids on the endothelial cytoskeleton via CD36/Cav-1-mediated endocytosis of oxLDL. In addition, several recent studies found that an increase in endothelial stiffness promotes monocyte adhesion and extravasation through the endothelial monolayer^30,34,35^, which is another major contributor to vascular dysfunction. Taken together, these observations suggest endothelial stiffening that develops in athero-prone regions of the aorta and during vascular ageing is an important contributor to chronic inflammation which is key to the gradual decline of vascular function.

## Supplementary Information

Supplementary Figures.

## Data Availability

The data that supports the finding of this study are available from the corresponding author upon request.
