# Supplementary material for: Caveolin-1 is a primary determinant of endothelial stiffening associated with dyslipidemia, disturbed flow, and ageing
Source: Sci Rep. 2022 Oct 24;12:17822. doi: 10.1038/s41598-022-20713-7 (PMC9592578; doi:10.1038/s41598-022-20713-7)
Supplement: Supplementary file 1 — Supplementary Figures. [file 41598_2022_20713_MOESM1_ESM.pdf]

Supplemental Figures and Captions

Supplemental Figure I: Endothelial alignment to laminar flow observed in WT but not Cav-1KO cells. EC alignment to the direction of laminar flow in WT (top) and Cav-1 KO MMVECs (bottom) following 48 hours of shear stress in the presence oxLDL (20-40 cells per condition/experiment, n=6-8). Scale bars represent 20 μm.

Supplementary Figure II: Differential flow patterns do not induced changes to endothelial stiffness. (A) Histograms and **(B)** averaged elastic modulus values for WT and Cav-1 KO MMVECs following 48 hours of laminar and disturbed flow in the absence of oxLDL.

Supplementary Figure III: Age-associated increase in aortic stiffening is Cav-1 dependent. Histograms of elastic modulus recorded from aortic arch of moderately aged (10-12 months, left column) and advanced aged (20-24 months, right column) WT (top) and Cav-1 KO (bottom) mice (n=5-6 mice, 10-20 measurements per sample).

Supplementary Figure IV: Endothelial stiffening in AA is independent of sex in WT mice. (A) Histograms and **(B)** summarized elastic modulus values from male and female 5-6 month old WT mice (experiments done in parallel, n=4, 10-15 tissue sites per condition per mouse). *$P<0.05$ compared to DA of same sex, analysis completed with ANOVA.

Supplemental Figure IV: Sub-endothelial stiffness in moderately and aged WT and Cav-1 KO mice. Elastic modulus values of the descending aorta and aortic arch regions from the intact sub-EC layer in moderately aged (10-12 month old) and advanced aged (20-24 month old) WT and Cav-1 KO mice (horizontal line depicts mean of n=5-6 mice with 10-15 measurements per sample). *$P<0.05$.

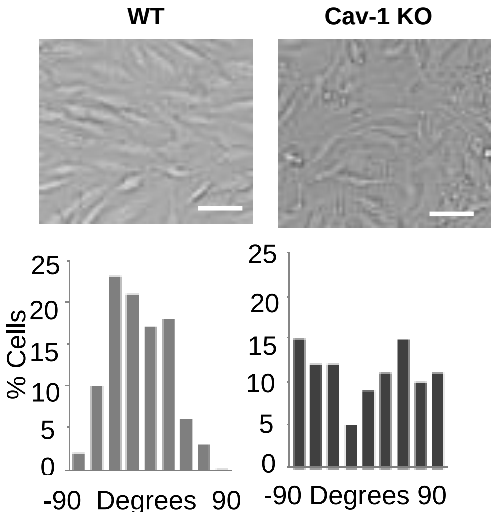

Supplemental Figure I

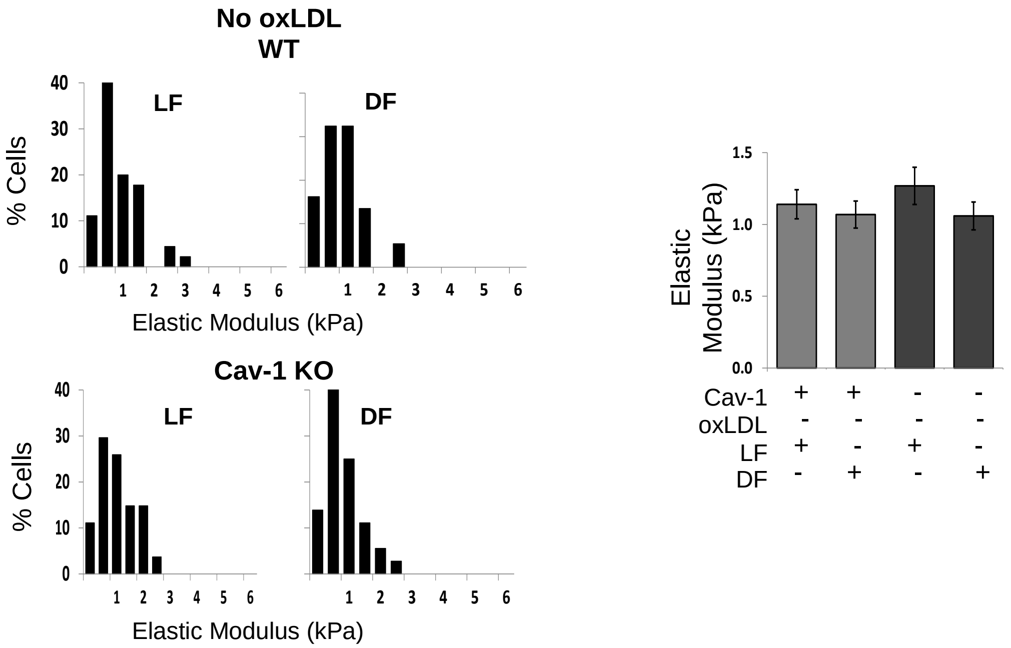

Supplemental Figure II

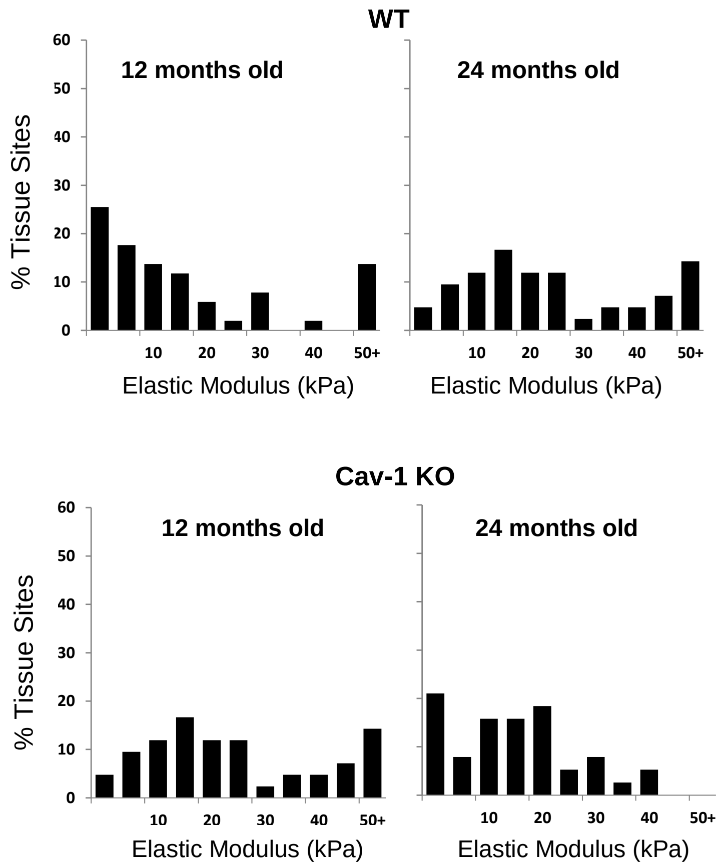

Supplemental Figure III

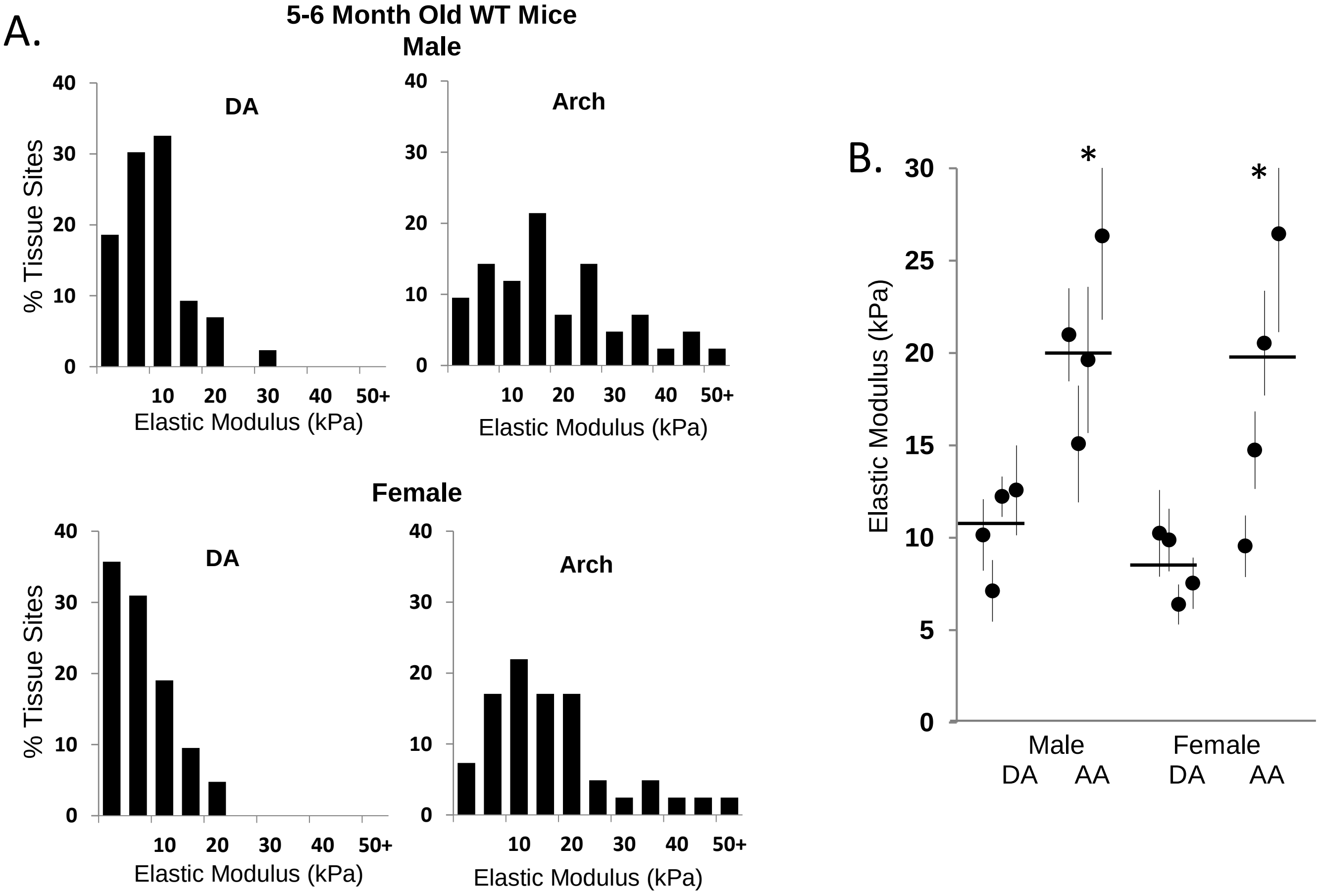

Supplemental Figure IV

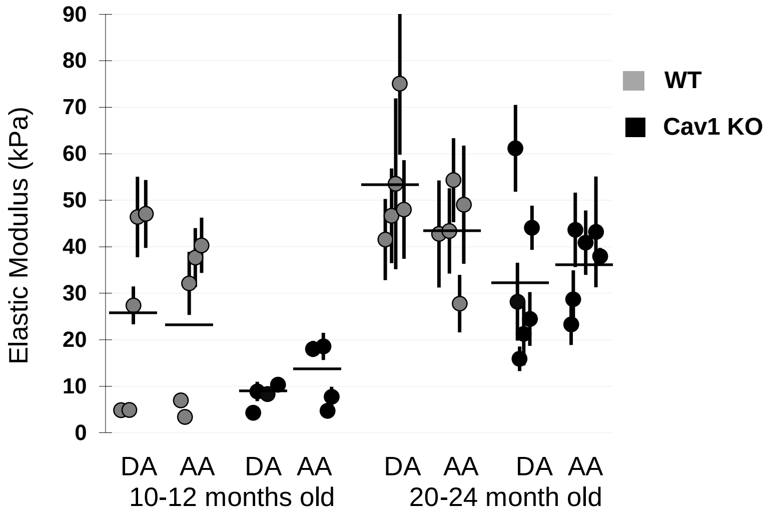

Supplemental Figure V
